# Optimizing Operating Room Efficiency for Primary Hip and Knee Arthroplasty Using Performance Benchmarks

**DOI:** 10.1016/j.artd.2024.101590

**Published:** 2024-12-24

**Authors:** Koorosh Kashanian, Matey Juric, Tim Ramsay, Pascal Fallavollita, Paul E. Beaulé

**Affiliations:** a Division of Orthopaedic Surgery, Department of Surgery, The Ottawa Hospital, University of Ottawa, Ottawa, Ontario, Canada; b Interdisciplinary School of Health Sciences, University of Ottawa, Ottawa, Ontario, Canada; c School of Engineering and Computer Science, University of Ottawa, Ottawa, Ontario, Canada

**Keywords:** THA, TKA, Efficiency, Benchmark

## Abstract

**Background:**

With increasing demand for total hip arthroplasty (THA) and total knee arthroplasty (TKA), maximizing operating room (OR) efficiency is critical. This paper sought to examine the implementation of time benchmarks when performing primary TKA and THA. We hypothesized that implementing benchmarks would improve efficiency and the number of joints performed per day.

**Methods:**

Two hundred ninety-five patients from 59 OR days were reviewed. All surgeries were performed as outpatients and included 160 females and 135 males; 161 TKA and 134 THA; mean age, 66.1; mean body mass index, 28.6 kg/m^2^; American Society of Anesthesia, 2. Time points, demographics, and adverse events were recorded. Benchmarks to complete 4 joints in 8 h were: anesthesia preparation time (APT) of <11 min, procedure time of <72 min, anesthesia finish time (AFT) of <21 min, and turnover of <22 min.

**Results:**

The percentage of cases meeting individual benchmarks for APT was 50.17%; procedure time was 95.25%; AFT was 99.67%; turnover was 65.25%. The means were: APT 11:00 min, Surgical Prep Time 9:00 min, procedure time 55:00 min, AFT 3:00 min, and turnover 19:00 min. Overall, 98.3% (58/59) of ORs had 4 cases completed within 8 h and 52.5% (31/59) had 5 cases within 8 h. Age, body mass index, and consecutive laterality of surgery were determined to affect the likelihood of meeting benchmarks for case time, APT, and turnover.

**Conclusions:**

Establishing time benchmarks permitted the introduction of 5 joint days within an 8-h OR without increasing resource utilization. Factors that influence OR efficiency for high-volume primary hip and knee replacements were identified.

## Introduction

There is an ever-rising demand for total hip arthroplasty (THA) and total knee arthroplasty (TKA). The population is continuing to age, and obesity is more prevalent than ever, increasing the burden of hip and knee osteoarthritis (OA). [[1], [2], [3]] In the United States, it is projected that by 2040 the demand for THAs and TKAs will increase to 1.43 million and 3.42 million annual procedures, respectively. To meet the demands of the population, a substantial increase in the current amount of procedures being done annually needs to occur. [4] However, rising overhead costs and decreased reimbursement place pressure on arthroplasty programs and demand maximized operating room (OR) efficiency and throughput. [[5], [6], [7], [8]] High overhead costs are limiting, with some systems instituting fewer OR days as a result. [9,10] As such, it is logical to maximize efficiency to minimize these increases in costs, performing more cases per operative day. This focus on increased efficiency involves 2 key pillars. Firstly, programs need to optimize procedures and perisurgical activity to limit case time and ensure patients progress as fast as safely possible. [5,11] Secondly, programs need to ensure that performance is tracked within their institution, allowing for evidence-based changes to be made in workflow, optimizing the various stages of the procedure from preop to recovery. Combined, the focus on optimizing workflow and tracking progress, allows for sustained and accelerated performance improvement.

Currently, many programs target 4 joints being operated within an 8-h shift in one OR. However, there are significant challenges in performing these within an 8-h shift as well as optimizing efficiencies to increase the number of joints per day. [12,13] One possible solution is the introduction of time benchmarks to allow better planning of each key step of the patient’s surgical journey, setting goals for all team members, and improving predictability for finishing on time. [14]

The purpose of this paper was to examine the implementation of time benchmarks for various phases of surgical care when performing primary THA and TKA. We hypothesized that establishing benchmarks would improve efficiency as well as throughput.

## Material and methods

After obtaining institutional review board approval, all THA and TKA performed from February to October 2023 at a stand-alone ambulatory arthroplasty center were reviewed. This period included 295 patients undergoing THA or TKA individually performed by 6 fellowship-trained arthroplasty surgeons with a single team of nurses and 1 anesthesiologist using 1 surgical suite. The patients’ mean age and body mass index (BMI) were 66.2 ± 8.6 year (range 32-88) and 28.62 ± 5.6 kg/m^2^ (range 16.9-52.0), respectively. The mean American Society of Anesthesia score was 2 (interquartile range 2-3) with 161 TKA (54.6%) and 134 THA (45.4%). There was a slightly higher percentage of female patients (n = 160, 54.2%) (Table 1).

**Table 1:** Demographic characteristics of cohort.

Parameter	Mean ± SD		
	Overall cohort (295)	Hips (134, 45.4%)	Knees (161, 54.6%)
Age	66.16 (±8.6)	63.40 (±9.49)	68.45 (±10.27)
Gender	Female: 160 (54.2%)	Female: 68 (50.8%)	Female: 92 (57.1%)
	Male: 135 (45.8%)	Male: 66 (49.2%)	Male: 69 (42.9%)
BMI	28.5 (±5.8)	27.43 (±8.3)	29.7 (±9.4)
ASA (mean)	2	2	2

ASA, American Society of Anesthesia.

**Figure 1:**
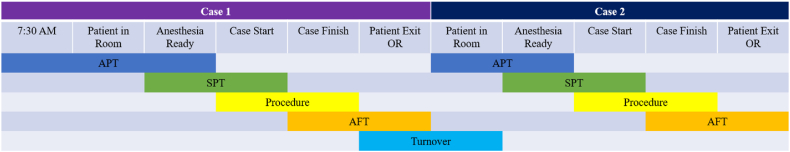
Time intervals during a 5-joint OR day.

Perioperative timeline information was extracted from the Surgical Information Management System in minutes examining well-established time points: 1) Anesthesia preparation time (APT): time from the patient entering the OR to when the anesthesiologist has prepped the patient for surgery; 2) Surgical prep time (SPT): Time from anesthesia ready to the case starting; 3) Procedure: case start to case finish; 4) Anesthesia finish time (AFT): case finish to patient leaving the OR; and 5) Turnover: patient exiting the OR to the next patient entering the OR (Fig. 1). [15] Total case time was measured from the patient entering the room to leaving the room.

All the members of the surgical team were gathered to discuss previously published benchmarks by Al-Zoubi et al. for each time point (Table 2) emphasizing a team approach to all phases of care. The purpose of these discussions was to provide an achievable standard and then measure to determine if this standard could be met. There was a focus on implementing 3 recommendations to achieve this. Recommendations were 1: Take time as a team at the beginning of the operative day to go over the slotted cases, 2: Utilize nursing teams who have received total joint training and are well-versed in arthroplasty workflow, 3: Ensure the surgical team is fully present for each case from positioning to patient transfer from the operative table. [12,16]

**Table 2:** Efficiency performance in the operating room theater.

Time point	Al Zoubi benchmark (mean)	Current study mean ± SD	Success in achieving benchmarks
Anesthesia prep time (APT)	<11 min	11.49 ± 7.12 min	50.17% (148/295)
Surgical preparation time (SPT)	-	9.93 ± 6.26 min	
Procedure time	<72 min	55.95 ± 19.92 min	95.25% (281/295)
Anesthesia finish time (AFT)	<21 min	3.96 ± 3.05 min	99.67% (294/295)
Turnover	<22 min	20.63 ± 12.26 min	62.25% (154/236)

SD, standard deviation.

The surgical days were built with 10-hour shifts to maximize staff engagement and savings through lowering of instrumentation mass (SLIM) instrument sets were used intraoperatively to reduce APT and procedure time, respectively. [17] SLIM instrument sets are standardized sets which have been produced in previous research to reduce waste and enhance efficiency. [17] All team members received the full pay for the shift and left when the surgical slate was complete, giving the incentive to finish as soon as possible to leave early with full pay. If time went over the slotted 10 hours, overtime pay was implanted. No block or flip rooms were used.

### Perioperative protocol

All patients received multimodal anesthesia of celecoxib for 1 week preoperatively and celecoxib and acetaminophen on the surgical date. There was a list of patients available on notice, should any cancellations occur last minute. Proceeding forth, spinal blocks were conducted in the OR by a staff anesthesiologist. hypobaric lidocaine was the agent used in all cases. [7] At the time of incision, patients received standard antibiotic prophylaxis with a weight-based dosage of cefazolin. All patients also received 1 g of tranexamic acid. Most THAs had an anterior approach in the supine position with the use of either positioning or standard table based on surgeon preference, while TKAs were performed using the medial parapatellar approach. There was no specific order regarding which cases (TKA vs THA) were performed first on days where both occurred in the same room.

At the time of component implantation, local infiltration analgesia consisting of a mix of ropivacaine, epinephrine, and ketorolac in saline was used based on patient weight. Patients were readministered tranexamic acid, antibiotics, and a multimodal analgesic regimen of celecoxib, acetaminophen, and tramadol in the postanesthesia recovery unit. Physiotherapists assisted in the care of all patients encouraging immediate weight-bearing to progress recovery.

### Statistical analysis

Demographics were reported using mean ± standard deviation or mean and interquartile range for continuous variables and as frequency and percentage for categorical variables. *t*-tests were used to compare age and BMI in cases where benchmarks were met vs when they were not. Fisher's exact test was applied to determine if there was any association between the laterality of consecutive surgeries being the same and benchmarks being met. All analyses were done using SAS version 9.4 for Windows (SAS Institute Inc., Cary, NC).

## Results

In total, the benchmarks were achieved in 78.2% (877/1121) of instances and individually they were met in 50.17% (148/295) of cases for APT, 95.25% (281/295) for procedure time, 99.67% (294/295) for AFT, and 65.25% (154/236) for turnover. All benchmarks were met for an individual case 32.2% (95/295) of the time, with all benchmarks in all cases for that OR day being met in 11.9% (7/59) of dates (Table 2).

The actual mean time points achieved were 11:00 min ± 7.12 min (min 0 minute, max 41 min) for APT, 9:00 min ± 6.26 min (min 0 min, max 33 min) for SPT, 55:00 min ± 19.92 min (min 5 min, max 153 min) for procedure, 3:00 min ± 3.05 min (min 0 min, max 28 min) for AFT, and 19 min ± 12.26 min (min 2 min, max 42 min) for turnover (Table 1). The mean time to complete 4 cases was 385 min (6 h and 25 min ± 46.92 min (Table 3) with 98.3% (58/59) within 8 h.

**Table 3:** Mean time intervals.

Time point	Mean ± SD
Total case time	95.13 (1:35) ± 26.30 min
Total time for 4 cases	385.02 (6:25) ± 46.92 min
Total time for 5 cases	472.33 (7:52) ± 52.81 min

SD, standard deviation.

**Figure 2:**
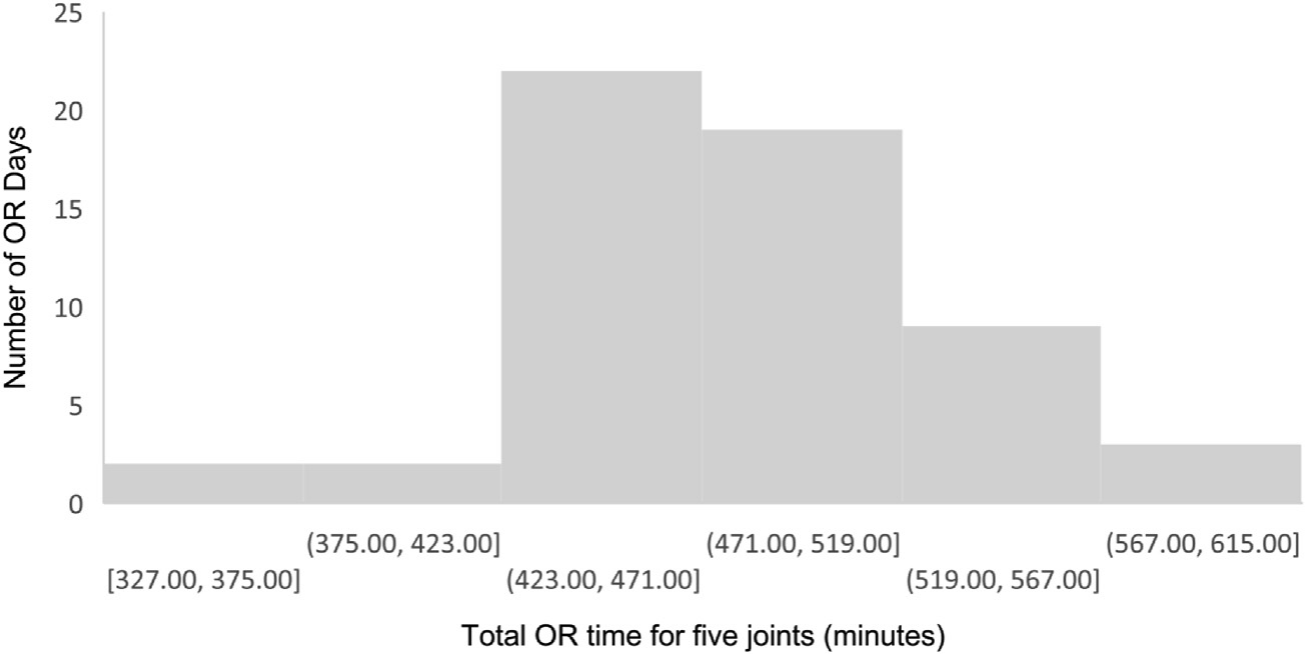
Distribution of total OR time for 5 joints.

The mean time of completion of 5 cases was 472 min ± 49.00 min (7 h and 52 min) (Fig. 2) with 52.5% (31/59) completed within 8 h and with 6 d missing the 8-h mark by 10 min or less. All 59 d finished all 5 cases 100% of the time within 10 h. New benchmarks were generated using the 59 surgery dates (Table 4) to explore the capacity of completing 5 joints within 8 h. Using the 70th percentile benchmarks, OR teams would complete their fourth and fifth case within 319 min (5 h and 19 min) and 403 min (6 h and 43 min), respectively. Following the same benchmarks at the 84th percentile, teams would complete their fourth and fifth case within 269 min (4 h 29 min) and 340 minutes (5 h 40 min), respectively.

**Table 4:** Benchmarks for 5 joint rooms.

Time point	Mean with 5 joints >480 min	Mean with 5 joints <480 min	70^th^ percentile	84th percentile
Anesthesia prep time (APT)	11.6 min	10.8	8 min	6 min
Surgical preparation time (SPT)	10 min	8 min	7 min	5 min
Procedure time	61.8 min	54.4 min	49 min	43 min
Anesthesia finish time (AFT)	4 min	3.6 min	3 min	2 min
Turnover	21.5 min	17.5 min	17 min	15 min
Total case time	103 min	92 min	85 min	76 min
Total time for 4 cases	415 min	364 min	365 min	352 min
Total time for 5 cases	516 min	454 min	458 min	442 min

Using the mean time points in Table 4, the mean overall case time was 95.13 (1:35) ± 26.30 min (Table 3) with 95.25% (281 out of 295) cases meeting that benchmark. Older patients (66.5 ± 8.6 y old vs 60.8 ± 6.9, *P* = .0052) and higher BMIs (31.2 ± 6.9 kg/m^2^ vs 28.4 ± 5.4 kg/m^2^, *P* = .0362) were associated with missing the total case time benchmark but not with the individual benchmarks (APT, SPT, procedure, AFT, turnover).

Cases were also analyzed to see if times were faster when it was the second consecutive case to be performed on the same side (left or right). In these same laterality cases, the APT benchmark was significantly more likely to be met, with 61% of same laterality cases meeting the APT benchmark, while 44% of cases which were not consecutive same laterality cases met that benchmark (*P* = .0074). This was a similar finding with turnover as well, with 70% of same laterality cases meeting the turnover benchmark and 51% of different-sided cases meeting the same benchmark (*P* = .0047). Other time points were not affected.

## Discussion

As overhead costs continue to increase, maximizing throughput without increasing resources ensures efficient use of OR time as well as an effective means to control overhead costs. [18] To be efficient in the OR, groups need to minimize the time spent performing activities not geared toward the patient as well as maximizing teamwork. However, this needs to be balanced with the safety of the team and patient, which are of the utmost priority. Establishing time performance indicators can facilitate gains in efficiency by providing a framework for discussion and areas of improvement. [19] In this consecutive case series, 98.3% of operating days had 4 cases completed within 8 hours with an average time of 385 min (6 h and 25 min) ± 46.92 min. All 5 joints were completed within 8 h in 52.5% (31/59) of days with a mean total time for a 5-joint day being 472 min ± 49.00 min (7 h and 52 min) (Fig. 2). These results show the implementation of time benchmarks in the OR theater through a collaborative approach is a cost-effective method of increasing performance while maintaining high quality. [20,21]

Although literature exists for benchmarking OR times, currently no uniform description of ‘optimization’ has been adopted. Other studies have looked at how operating times differ between academic and community hospitals and found that the anesthesia control time (analogous to this study’s APT definition) was approximately 4 min longer in academic medical centers compared to community hospitals for both TKA and THA. [14] Additionally, the surgery control time (analogous to this study’s procedure time definition) was approximately 11 and 14 min longer for THA and TKA, respectively, in academic medical centers. However, this study is the first to look at the feasibility of adding an extra procedure per 8-h OR list because of gained efficiencies by establishing benchmarks for the various intraoperative time stamps. These results may have significant implications for the overall cost per case in the era of bundle care. [22] Some of those costs have been significantly mitigated by the introduction of day surgery programs for hip and knee replacements. [9] When comparing the average hospital cost of a day surgery vs inpatient the cost savings are significant, ranging from 20%-35%. [9,23] In addition, day surgery programs at ambulatory surgical centers promise more efficient day-to-day operations, with similarly positive patient outcomes compared to larger hospital systems. Focusing on achieving 5-joint day surgery arthroplasty programs is an additional method to build upon these improvements, particularly in independent ambulatory surgical centers, and decrease health-care expenditure by substantial amounts.

**Figure 3:**
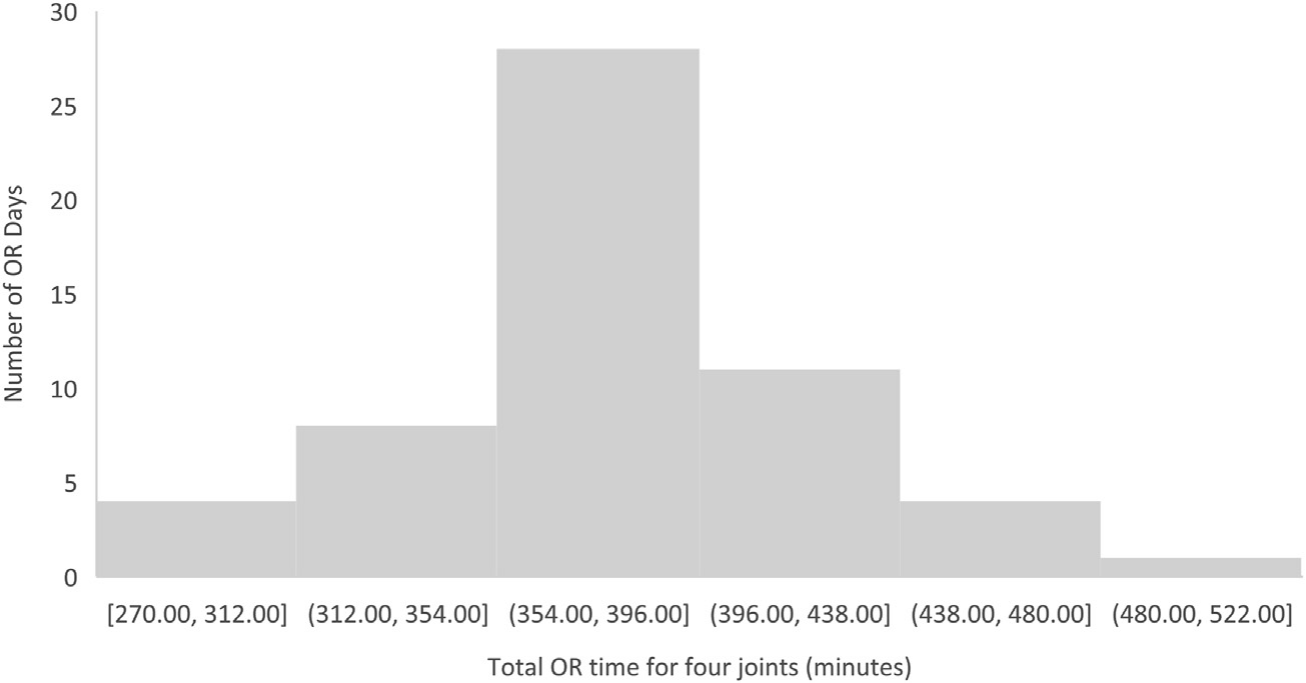
Distribution of total OR time for 4 joints.

Previously published success rates for performing 4 joints within 8 h have been reported as low as 39%, which is in sharp contrast to the current results where 98.3% (58/59) of 4-joint days finished within 8 h, and 52.5% (31/59) of 5-joint days finished within 8 hours. [5] One possible explanation is that the sharing of published benchmarks with the members of the surgical team as well as the introduction of SLIM sets enhanced the focus of our team toward achieving those benchmarks. If mean benchmarks can be met by members of the OR teams, a typical 4-joint day can be converted to add a fifth case within a standard 8-h OR period in just over 400 min (Fig. 3). To further improve performance, more lean benchmarks could be considered as shown in Table 4, specifically focusing on the 70th percentile, or 1 standard deviation above the mean, at the 84th percentile. However, there must be a balance between patient safety and the team’s comfort level. Consequently, one may want to consider focusing improvement on specific benchmarks such as turnover where a benchmark of 13 min vs 17 min would represent 16 min of saved time without impacting patient safety. While other time points such as the procedure time, and to some extent APT, could also be improved, they may be more challenging as they are more dependent on the performance of one team member compared to the whole team. In addition, other factors were found to impact total case time which can be somewhat controlled such as age and BMI of the patient, as well as the order of cases, particularly whether multiple procedures on the same side occur in succession, all of which significantly impacted surgical performance. These are factors which could be managed ahead of time in the consideration of 5-joint day arthroplasty programs to enhance efficiency. In other words, limiting the number of patients with high BMI within a surgical list and/or older patients would ensure a balanced mix concerning potential impact on both procedure time and APT where difficulties with intravenous access and/or surgical dissections, as well as patients being more frail or heavier to transfer, would lead to longer days. [24] There was also an interesting finding of which the literature is sparse. In particular, it was discovered that there is a positive effect on OR efficiency with successive surgeries being performed on the same side where APT and turnover were both demonstrated to be significantly lower when the preceding case was on the same side. This highlights the importance of optimized workflows in OR efficiency.

There are several limitations of this study, such as the retrospective nature in which the data were reviewed, which introduces biases in patient selection or categorization on different days and could have affected performance measurements. However, there were no outliers noted on the analysis of different OR dates, so this is less likely. In addition, the analysis which revealed that factors such as age, BMI and consecutive laterality of surgery could affect the ability of the team to meet benchmarks was correlative. Another limitation is the specific work environment and staff expertise which may not be reproducible in a different institution. For example, there were no block rooms used which have been shown to dramatically improve OR efficiency, hence it is unclear how these benchmarks would apply in that type of setting. In addition, teams were incentivized via fee-for-service to ensure all team members were motivated to increase efficiency and throughput, which may not be achievable in all practices, namely academic practices. However, the given benchmarks and time points are relevant in demonstrating the optimization of time points and can provide values to compare institutional performance. Further research should be conducted to determine how exactly these factors affect time points and what can be done to mitigate their effects on operative workflow.

## Conclusions

Using time benchmarks, a 98.3% (58/59) success rate was achieved in completing 4 joints within 8 h and a 52.5% (31/59) success rate in finishing 5 joints in the same timeframe. When examining higher-performing benchmarks such as the 70th percentile or 84th percentile, 5 joints could be predictably done in a high percentage of cases within 8 h while trying to optimize patient-related factors such as BMI, age, and consecutive laterality to further optimize OR efficiency. Overall, this study highlights that the implementation of benchmarks in high-volume primary joint arthroplasty leads to improvements in operating efficiency and throughput.

## Conflicts of interest

P.E. Beaulé receives royalties from Corin, MicroPort, Medacta, MatOrtho, is a DePuy Synthes, MicroPort, MatOrtho, Zimmer Biomet consultant, receives research support Zimmer Biomet, MicroPort Orthopedics, Medacta, Corin, receives royalties, financial or material support from Wolters Kluwer, and is an International Society for Hip Arthroscopy (ISHA) board member; all other authors declare no potential conflicts of interest.

For full disclosure statements refer to https://doi.org/10.1016/j.artd.2024.101590.

## CRediT authorship contribution statement

**Koorosh Kashanian:** Writing – review & editing, Writing – original draft, Visualization, Validation, Project administration, Methodology, Investigation, Formal analysis, Data curation, Conceptualization. **Matey Juric:** Writing – review & editing, Writing – original draft, Validation, Software, Formal analysis, Data curation. **Tim Ramsay:** Writing – review & editing, Formal analysis, Data curation. **Pascal Fallavollita:** Writing – review & editing, Formal analysis. **Paul E. Beaulé:** Writing – review & editing, Visualization, Validation, Supervision, Software, Resources, Project administration, Methodology, Investigation, Funding acquisition, Data curation, Conceptualization.

## Appendix A Supplementary data

Conflict of Interest Statement for Kashanian

Conflict of Interest Statement for Matey

Conflict of Interest Statement for Beaulé

Conflict of Interest Statement for Fallavollita

Conflict of Interest Statement for Ramsay
