# Supplementary material for: Optimizing Operating Room Efficiency for Primary Hip and Knee Arthroplasty Using Performance Benchmarks
Source: Arthroplast Today. 2024 Dec 24;31:101590. doi: 10.1016/j.artd.2024.101590 (PMC11732218; doi:10.1016/j.artd.2024.101590)
Supplement: Conflict of Interest Statement for Beaulé [file mmc3.pdf]

INDIVIDUAL CONFLICT OF INTEREST STATEMENT

American Association of Hip and Knee Surgeons

(Adopted from the American Academy of Orthopaedic Surgeons disclosure statement)

The following form **must be filled out completely and submitted by each author (example, 6 authors, 6 forms). All items require a response. If there is no relevant disclosure for a given item, enter "*None*."**

Manuscript Title: Optimizing Operating Room efficiency for Primary Hip and Knee Arthroplasty using performance benchmarks

1. Royalties from a company or supplier (The following conflicts were disclosed)

Corin, MicroPort, Medacta, MatOrtho

2. Speakers bureau/paid presentations for a company or supplier (The following conflicts were disclosed)

None

3A. Paid employee for a company or supplier (The following conflicts were disclosed)

None

3B. Paid consultant for a company or supplier (The following conflicts were disclosed)

DePuy Synthes, MicroPort, MatOrtho, Zimmer Biomet

3C. Unpaid consultants for a company or supplier (The following conflicts were disclosed)

None

4. Stock or stock options in a company or supplier (The following conflicts were disclosed)

None

5. Research support from a company or supplier as a Principal Investigator (The following conflicts were disclosed)

Zimmer Biomet, MicroPort Orthopedics, Medacta, Corin

6. Other financial or material support from a company or supplier (The following conflicts were disclosed)

None

7. Royalties, financial or material support from publishers (The following conflicts were disclosed)

Wolters Kluwer

8. Medical/Orthopaedic publications editorial/governing board (The following conflicts were disclosed)

None

9. Board member/committee appointments for a society (The following conflicts were disclosed)

International Society for Hip Arthroscopy (ISHA)

Each author must sign AND print or type his/her name, date and submit a separate form

In addition, one BLINDED Conflict of Interest form (no author names used) should be submitted per manuscript with all author disclosures.

Paul Beaulé	[signature]	15-Feb-2024
Author Name (Print or Type)	Author Signature	Date
