# Supplementary material for: Optimizing Operating Room Efficiency for Primary Hip and Knee Arthroplasty Using Performance Benchmarks
Source: Arthroplast Today. 2024 Dec 24;31:101590. doi: 10.1016/j.artd.2024.101590 (PMC11732218; doi:10.1016/j.artd.2024.101590)
Supplement: Conflict of Interest Statement for Ramsay [file mmc5.pdf]

INDIVIDUAL CONFLICT OF INTEREST STATEMENT

American Association of Hip and Knee Surgeons

(Adopted from the American Academy of Orthopaedic Surgeons disclosure statement)

The following form **must be filled out completely and submitted by each author (example, 6 authors, 6 forms). All items require a response. If there is no relevant disclosure for a given item, enter "*None.*"**

Manuscript Title: Optimizing Operating Room efficiency for Primary Hip and Knee Arthroplasty using performance benchmarks

1. Royalties from a company or supplier (The following conflicts were disclosed) **No**

2. Speakers bureau/paid presentations for a company or supplier (The following conflicts were disclosed) **No**

3A. Paid employee for a company or supplier (The following conflicts were disclosed) **No**

3B. Paid consultant for a company or supplier (The following conflicts were disclosed) **No**

3C. Unpaid consultants for a company or supplier (The following conflicts were disclosed) **No**

4. Stock or stock options in a company or supplier (The following conflicts were disclosed) **No**

5. Research support from a company or supplier as a Principal Investigator (The following conflicts were disclosed) **No**

6. Other financial or material support from a company or supplier (The following conflicts were disclosed) **No**

7. Royalties, financial or material support from publishers (The following conflicts were disclosed) **No**

8. Medical/Orthopaedic publications editorial/governing board (The following conflicts were disclosed) **No**

9. Board member/committee appointments for a society (The following conflicts were disclosed) **No**

Each author must sign AND print or type his/her name, date and submit a separate form

In addition, one BLINDED Conflict of Interest form (no author names used) should be submitted per manuscript with all author disclosures.

Tim Ramsay — Tim Ramsay — July 17, 2024

Author Name (Print or Type) — Author Signature — Date
